# Application of β-Tricalcium Phosphate in Adhesive Dentin Bonding

**DOI:** 10.3390/polym13172855

**Published:** 2021-08-25

**Authors:** Mohammad H. AlRefeai, Eman M. AlHamdan, Samar Al-Saleh, Abdulaziz S. Alqahtani, Mohammad Q. Al-Rifaiy, Ibraheem F. Alshiddi, Imran Farooq, Fahim Vohra, Tariq Abduljabbar

**Affiliations:** 1 Department of Restorative Dentistry—Operative Division, College of Dentistry, King Saud University, P.O. Box 21069, Riyadh 11475, Saudi Arabia; malrefeai@ksu.edu.sa; 2 Department of Prosthetic Dental Science, College of Dentistry, King Saud University, P.O. Box 21069, Riyadh 11475, Saudi Arabia; ealhamdan@ksu.edu.sa (E.M.A.); Salsaleh@ksu.edu.sa (S.A.-S.); absalqahtani@ksu.edu.sa (A.S.A.); dean.alrifaiy@gmail.com (M.Q.A.-R.); ialshiddi@ksu.edu.sa (I.F.A.); fvohra@ksu.edu.sa (F.V.); 3 Faculty of Dentistry, University of Toronto, Toronto, ON M5G 1G6, Canada; imran.farooq@mail.utoronto.ca

**Keywords:** dentin, adhesive, calcium, phosphate, tricalcium phosphate

## Abstract

The study aimed at synthesizing β-tricalcium phosphate (β-TCP) nanoparticles and comparing the mechanical properties and dentin interaction of two adhesives: experimental adhesive (EA) and EA with 5 wt.% β-TCP nanoparticles (β-TCP-5%). These filler nanoparticles were synthesized and then characterized with scanning electron microscopy (SEM) and micro-Raman spectroscopy. The β-TCP nanoparticles were incorporated in the adhesives to form two groups: gp-1: EA (control) and gp-2: β-TCP-5%. These adhesives were characterized by SEM, energy-dispersive X-ray (EDX) spectroscopy and were also assessed for their micro-tensile bond strength (μTBS) with (TC) and without thermocycling (NTC). Fourier Transform Infrared (FTIR) spectroscopy was performed to evaluate the degree of conversion (DC) of two adhesives. The β-TCP filler was seen as irregularly shaped agglomerates on SEM. The micro-Raman spectra revealed characteristic peaks associated with β-TCP nanoparticles. Both adhesives presented suitable dentin interaction, which was demonstrated by the formation of resin tags of variable depths. The EDX analysis verified the existence of calcium (Ca) and phosphate (P) for the β-TCP-5% group. The greatest μTBS values were shown by β-TCP-5% group samples when they were non-thermocycled (NTC) (β-TCP-5%-NTC: 34.11 ± 3.46) followed by the thermocycled (TC) samples of the same group (β-TCP-5%-TC: 30.38 ± 3.66), compared with the EA group. Although the DC presented by β-TCP-5% group was comparable to the EA group, it was still lower. The addition of β-TCP nanoparticles in the adhesive improved its μTBS and resulted in a suitable dentin interaction, seen in the form of hybrid layer and resin tag formation. Nonetheless, a decreased DC was observed for the β-TCP-5% adhesive. Future studies probing the effect of different filler concentrations on various properties of the adhesive are warranted.

## 1. Introduction

The clinical success of bonded restorations is immensely reliant on the role played by dentin adhesives [1]. The bond formed by the adhesive with the tooth tissue is unfortunately unstable and loses strength over time [2]. The bond formed depends on the monomers’ ability to infiltrate the inter-collagen spaces in the dentin tissue to form steady resin tags and subsequently establish a stable hybrid layer [3]. Additionally, the bond durability not only depends on the structure of the tissue with which the adhesive is being bonded with (enamel or dentin) [4] but is also reliant upon various properties of the adhesives that are influenced by their composition [5]. Therefore, it has been recommended previously that the integration of bioactive inorganic fillers in the adhesive could augment the strength of this bond, preventing gradual loss in its strength [6,7]. Researchers have demonstrated encouraging results after the incorporation of various fillers in the adhesives that included filler particles based on bioactive glass (BG), hydroxyapatite (HA), graphene oxide (GO), silica (SiO_2_), calcium fluoride (CaF_2_), and zinc chloride (ZnCl_2_) [6]. The inclusion of these filler nanoparticles improves various properties of the adhesives, consequently improving longevity of the dental resin restorations [8]. One such category of fillers is based on tricalcium phosphate (TCP) nanoparticles. 

TCP is a resorbable material that has been used primarily for bone regeneration purposes [9]. Although many polymorphs exist, two polymorph phases (α and β) are primarily utilized as biomaterials [10]. In dentistry, both α-TCP and β-TCP possess wide-ranging clinical applications including (but not limited to) their use in a toothpaste to remineralize enamel subsurface lesions [11], in dental varnishes to reduce enamel demineralization [12], and in adhesives to improve their properties [13]. β-TCP was introduced in the early 1970s by Driskell and team primarily to treat bony defects [14]. β-TCP is a biocompatible material that is of particular interest for various clinical applications as it can be synthesized with consistent batch quality because it has well-defined properties [15]. In the last two decades, their use in dentistry has increased and β-TCP has shown its efficacy in many applications, especially in the vital pulp therapy as a direct pulp-capping agent [16,17], in treating periodontal infra-bony defects [18], and in alveolar ridge preservation for implant placement [19]. Schiroky et al. formerly incorporated TCP nanoparticles in their experimental adhesive (EA) and reported higher Knoop hardness and polymerization rate as compared with the controls [13]. Tavassoli-Hojjati et al. previously reported that the addition of 1–5% of β-TCP nanoparticles improves remineralization potential of the material [20]. Considering these advantageous properties of TCP nanoparticles, it was decided in our study to include 5 wt.% β-TCP nanoparticles in our EA as their incorporation could boost numerous mechanical properties of the adhesives. 

This study hypothesized that the inclusion of β-TCP nanoparticles would amplify adhesive’s bond strength, stability, and interaction with dentin tissue. The study aimed to produce and characterize a β-TCP containing EA with methods encompassing scanning electron microscopy (SEM)—line Energy Dispersive X-Ray (EDX) spectroscopy, micro-Raman spectroscopy, micro-tensile bond strength (μTBS) testing, Fourier-transform infrared (FTIR) spectroscopy, and a degree of conversion (DC) analysis.

## 2. Materials and Methods

The study received approval from the ethics committee of the institute before commencement, and all the ethical protocols mentioned in the Helsinki Declaration (1964 and its future modifications) were firmly trailed. 

### 2.1. Production of β-TCP Nanoparticles

We followed the earlier recommendations of Mehdikhani and Borhani [21] to synthesize β-TCP nanoparticles. Briefly, calcium nitrate tetra-hydrate ((Ca(NO_3_)_2_·4H_2_O) (98%, Merck, Kenilwork, NJ, USA) and diammonium hydrogen phosphate ((NH_4_)2HPO_4_) (99%, Merck) were reacted to produce β-TCP nanoparticles. Initially, a white precipitate was produced by adding 500 mL of 0.6 mol Ca(NO_3_)_2_ (pH = 7.3) dropwise over a time period of 2–3 h in 500 mL of 0.4 mol (NH_4_)2HPO_4_ solution (pH = 4) that was robustly agitated before the mixing. During this reaction, aliquots of 0.1 M sodium hydroxide (99%, Merck) were used to maintain the pH of the system at 8. The resultant white precipitate was first stirred for 12 h and then washed with distilled water (DW) and ethanol to augment its dispersion features. Utilizing mild suction, this mixture was filtered inside a filter glass. Post-filtration drying of the filtration cake was carried out at 80 °C for one day. On the next day, the dried powders were obtained and broken down using a mortar and pestle and calcined inside an alumina crucible at 700 °C temperature for 2 h.

### 2.2. Synthesis of the EA Containing β-TCP Nanoparticles

The EA was prepared by shadowing the steps of our earlier study [22]. For the EA’s formulation, a mixture of monomers consisting of bisphenol A glycol dimethacrylate (BisGMA), triethylene glycol dimethacrylate (TEGDMA), 2-hydroxyethyl methacrylate (HEMA), and ethyl 4-dimethylamino benzoate and camphorquinone (Esstech Inc., Essington, PA, USA) were utilized. Our EA composition involved 50%-Bis-GMA, 25%-TEGDMA, and 25%-HEMA (60%) by weight, with ethanol (30% m/m) utilized as a solvent. The photo-initiators in our EA included 0.5% (*n/n*) ethyl 4-dimethylamino benzoate and 0.5% camphorquinone that were included in accordance with the monomer moles. Furthermore, 1.0% (*n/n*) diphenyliodonium hexafluorophosphate (DPIHP) was included as an electron initiator to the adhesive mixture. This merger was prepared in a three-necked flask by means of a magnetic stirrer and a condenser (SA300; Sansyo, Tokyo, Japan). This new EA was isolated in a foil-sheltered dark compartment to escape photo-polymerization.

To incorporate the β-TCP nanoparticles in the newly synthesized EAs and achieve homogenization, these filler particles’ dispersion was performed using sonication in a centrifuge. These adhesives were kept stored at EAs with and without β-TCP nanoparticles at 4 °C and were used within 3 weeks of their preparation.

### 2.3. Evaluation of β-TCP Filler Nanoparticles 

The morphological characteristics of β-TCP nanoparticles were investigated utilizing SEM. A small quantity of filler nanoparticles was mounted on aluminum stubs, gold-coated, and viewed in an SEM (FEI Quanta 250, Scanning Electron Microscope, Plymouth, OR, USA), and an accelerating voltage of 30 kV was employed for these investigations. Different magnifications were used to image our study samples. 

The micro-Raman spectroscopy was implemented to assess β-TCP nanoparticles. A Raman spectrophotometer (ProRaman-L Analyzer; TSI, Shoreview, MN, USA) with a Raman reader^®^ software was employed to attain Raman spectra(s). The laser beam was secured through a 0.9 objective lens, and 600 mW power and 1 min scan were accomplished thrice.

### 2.4. Tooth Sample Preparation and Bonding Protocol

Sixty teeth (*n* = 60) were obtained post-extraction from the orthodontic clinics of the institution and then cleaned utilizing an ultrasonic scaler (Superior Instruments Co., New York, NY, USA). These teeth were then kept for two days in chloramine trihydrate solution (Merck, Germany). These teeth were then embedded in orthodontic resin (Opti-Cryl, South Carolina, Columbia) at the level of cementoenamel junction (CEJ) within 15 mm (height) sections of polyvinyl pipes (4 mm) and stored in DW. Dentin tissue of these teeth was exposed using a slow-speed diamond saw (Buehler Isomet 2000 Precision saw, Lake Bluff, IL, USA) that removed the coronal enamel. This dentin was exposed for 10 s to 35% phosphoric acid (Ultra etch Econo Kit-Optident, Yorkshire, UK), which was trailed by its washing and drying with DW and cotton pellets, respectively. These teeth were equally and randomly allocated to two groups (*n* = 30) (based on the adhesive they were treated with); gp-1: EA (without β-TCP nanoparticles, control), and gp-2: EA with 5% β-TCP nanoparticles (β-TCP-5%). For their treatment, the adhesives were layered onto the dentin surfaces for 10 s with a micro-brush used with agitation trailed by 3 s of air thinning. Another layer of adhesive was then smeared, and then polymerization was carried out for 20 s from a 10mm distance with the help of a light-curing device (Eliphar S10; 600 mW·cm^−2^ output; 3M ESPE, St. Paul, MN, USA). A resin composite incremental build-up (Filtek Universal; 3M ESPE, St. Paul, MN, USA) was achieved for each sample on the bonded adhesive utilizing a resin mold and metal condenser. The excess material was removed, and this build-up was polymerized using a curing device for 20 s. These bonded samples were stowed in DW for 1 day at 37 °C. Out of the thirty samples in each group, twenty were used for μTBS testing, five were utilized to assess bond integrity using SEM-EDX, and five were used for micro-Raman analysis. 

### 2.5. SEM-EDX Examination of the Bonded Adhesive-Dentin Interface

The bonded samples belonging to EA and β-TCP-5% groups were first polished (Beuhler Polisher, Lake Bluff, IL, USA) and then cleaned for 4 min in an ultrasonic bath (Bandelin Digital-Sigma-Aldrich Darmstadt, Germany). With the help of 35% phosphoric acid gel (Ultra etch Econo Kit-Optident-, Yorkshire, UK) application for 10 s, these bonded beams were treated at the interface and then washed for 15 s with DW. The specimens were then dipped for 5 min in a 5.25% sodium hypochlorite (NaOCl) solution and washed. Ethanol treatment at various concentrations (80%, 90%, and 100%) was executed to desiccate our study specimens. These were then observed under an SEM (FEI Quanta 250, Scanning Electron Microscope, OR, USA) with secondary electron mode of 10 kV by first mounting them on aluminum stubs which was followed by their gold coating. In our study, EDX was utilized to assess different elemental distributions inside the adhesive and hybrid layer. A line EDX was completed to exhibit the distribution of elements together with β-TCP-5% at the adhesive-dentin interface.

### 2.6. μTBS Analysis

Pre-sample sectioning, ten samples from each group were thermo-cycled (TC) with 10,000 cycles while the remaining ten remained non-TC (NTC, stored in DW for 1 week). The TC was carried out inside a water bath (THE-1100, SD Mechatronik GmbH, Berlin, Germany) containing separate water chambers at 5 °C and 55 °C. The samples were immersed for 30 s, whereas the dwell time was 5 s. A slow-speed diamond saw (Buehler Isomet 2000 Precision saw, Lake Bluff, IL, USA) was utilized to section the bonded samples to form composite-dentin bonded beams (1 mm × 1 mm). Individually, every tooth produced seven beams (seventy in each adhesive group) for μTBS evaluation (β-TCPA-5%-TC, β-TCPA-5%-NTC, EA-TC, and EA-NTC). These beams were fixed in the micro-tensile tester (Bisco Inc., Richmond, VA, USA) jaws using cyanoacrylate (Superglue, MN, USA). The assessment was carried out in tension at a crosshead speed of 0.5 mm/minute until failure. The nature of the failure modes in our study were also appraised and regarded as adhesive, cohesive, and mixed kinds employing a digital microscope (Hirox KH 7700, Tokyo, Japan). 

### 2.7. FTIR and DC Investigation

The DC of the adhesives was calculated by employing FTIR spectroscopy. The adhesives belonging to the two groups were analyzed before and after curing. Standardized adhesives were smeared on the potassium bromide discs of the spectroscope (Shimadzu, Kyoto, Japan). As the adhesives maintained contact with the FTIR sensors (Thermo Scientific Nicolet iS20 FTIR spectrometer, Waltham, MA, USA), absorbance peaks for the C–C double bonds were noted for the uncured resin. After the polymerization of the adhesive resin, FTIR peaks were recorded again. The C–C (aromatic) reference peaks (1607 cm^−1^) and C = C (aliphatic) absorbance peaks (1638 cm^−1^) were attained using an earlier standardized method [23]. To define the DC, spectra (s) were obtained in the range of 400 and 4000 cm^−1^. The DC was estimated by comparing the ratios of (C=C and C–C) absorbance strengths (% of unreacted double bonds) pre- and post-curing utilizing the following formula [24].

DC = 100% × [1 − (caliphatic/caromatic)/(ualiphatic/uaromatic)]

where caliphatic represents 1638 cm^−1^ absorption peak of cured resin, caromatic represents 1607 cm^−1^ absorption peak of cured resin, ualiphatic represents 1638 cm^−1^ absorption peak of uncured resin, and uaromatic represents 1607 cm^−1^ absorption peak of uncured resin.

### 2.8. Statistical Analysis 

The μTBS and DC analysis test values were statistically analyzed using SPSS-20.0 (IBM, Chicago, IL, USA). The ANOVA and Tukey’s post hoc multiple comparisons test were employed, and the level of significance was set at 1%. 

## 3. Results

### 3.1. Morphological Characteristics of the β-TCP Nanoparticles

**Figure 1:**
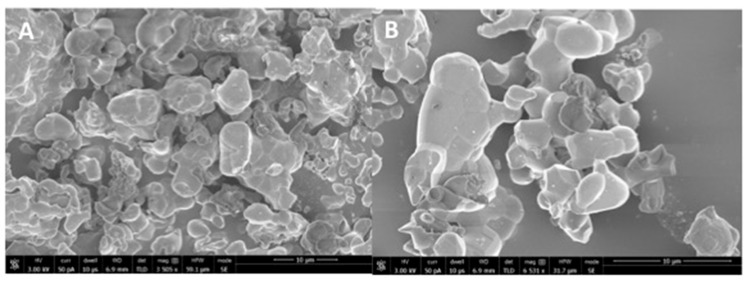
(**A**) Low (3505x) and (**B**) high magnification (6531x) SEM view of the synthesized nano β-tricalcium phosphate particles. The powder of β-tricalcium phosphate showed agglomerated morphology of various irregular sized polygonal crystals ranging from 500 nm to 1500 nm formed by sol-gel synthesis.

**Figure 2:**
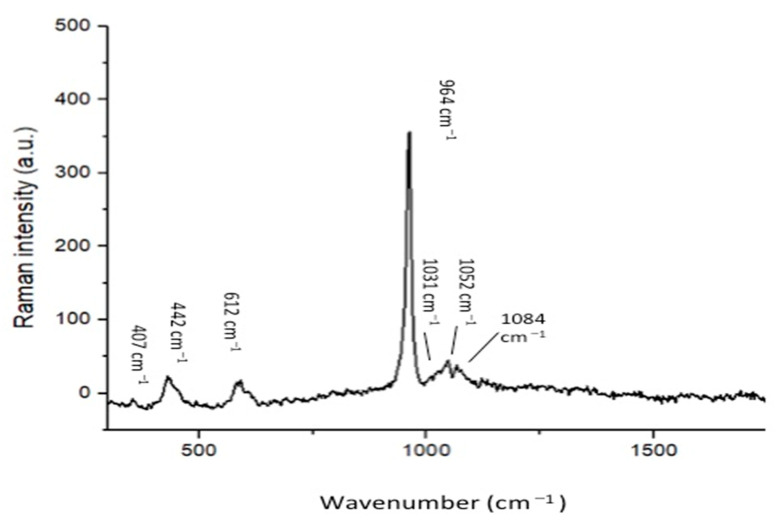
Raman spectrum of the nano-β-tricalcium phosphate indicates the labeled peaks as characteristic of the internal vibration of the PO_4_^3−^ tetrahedric groups of the β-TCP molecule. The symmetric stretching (ν_1_) of P-O bonds of the tetrahedron corresponds to the peaks with the highest intensity at around 950 cm^−1^ and 970 cm^−1^. The asymmetric stretching (ν_3_) has a lower intensity and is in the 1015–1090 cm^−1^ range.

The representative SEM micrographs of β-TCP nanoparticles are presented in Figure 1A (low magnification) and 1B (high magnification). The β-TCP nanoparticles were agglomerated and revealed irregular morphology (Figure 1A,B). The particle size range was 500–1500 nm. The representative micro-Raman spectra of the β-TCP nanoparticles is presented in Figure 2. In the spectra, symmetric (v_1_) and asymmetric stretching (v_3_) were both observed. The v_1_ of the P-O bonds of the tetrahedron relate to the peaks with the greatest intensities observed nearby 950–970 cm^−^^1^. These bands could arise because of the factor group splitting of the v_1_ fundamental vibrational mode matching with the symmetric P-0 stretching vibration of the phosphate ion. The v_3_ had less intensity and was found in the range 1015–1090 cm^−^^1^. The splitting of the v3 fundamental vibrational mode in β-TCP could have resulted in the formation of two bands seen at 1052 and 1084 cm^−^^1^ (Figure 2).

### 3.2. SEM-EDX Analysis Outcomes of the Bonded Adhesive-Dentin Interface

**Figure 3:**
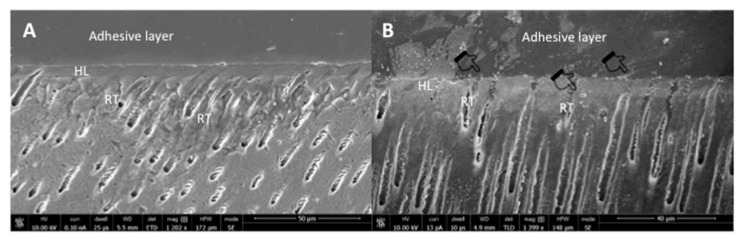
Representative SEM images of the bonded resin dentin interface using (**A**) unmodified dentin adhesive (EA) and (**B**) 5 wt.% nano-β-tricalcium phosphate modified dentin adhesive. Note the addition of nanocrystals (agglomerations in the image as indicated by pointers) did not significantly affect dentin bonding at the hybrid layer (HL) with well-formed resin tags (RT).

**Figure 4:**
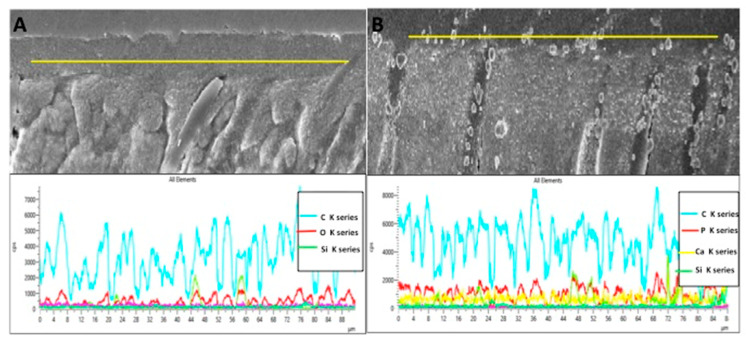
Representative line EDX along the resin dentin interface/hybrid layer for (**A**) unmodified dentin bonding agent (EA) and (**B**) 5 wt% nano-β-tricalcium phosphate modified dentin bonding agent. The modified dentin bonding agent indicates the presence of calcium (Ca), phosphorus (P), and other tooth related elements like silica filler (Si) and carbon (C).

The representative SEM micrographs demonstrating the dentin interaction of the EA and β-TCP-5% adhesive are presented in Figure 3A,B, respectively. Both adhesives showed suitable dentin interaction, which was demonstrated by the appropriate bonding and formation of resin tags of varying depths (Figure 3A,B). The representative line EDX of the EA and β-TCP-5% adhesive are presented in Figure 4A,B, respectively. Elements like carbon (C), oxygen (O), and silica (Si) were detected along the interface for the EA group (Figure 4A). For β-TCP-5%, in addition to the elements found in the EA, calcium (Ca) and phosphorus (P) were also witnessed, which confirmed the presence of β-TCP nanoparticles in our gp-2 (Figure 4B).

### 3.3. μTBS Analysis Outcomes

The outcomes of the μTBS analysis were computed as means ± standard deviations and are displayed in Table 1. The highest μTBS values were obtained for β-TCPA-5%-NTC (34.11 ± 3.46) followed by β-TCPA-5%-TC (30.38 ± 3.66) (Table 1). The EA group presented lower μTBS values (EA-NTC: 28.07 ± 2.60, EA-TC: 20.94 ± 3.37) as compared with the β-TCPA-5% adhesive. In general, it was observed that on TC, μTBS values of both the groups decreased (Table 1). Intra-group statistical comparisons revealed non-significant results (*p* > 0.01) for both adhesive groups. All inter-group statistical comparisons (β-TCPA-5%-NTC compared with EA-NTC and β-TCPA-5%-TC matched with EA-TC) were statistically significant (*p* < 0.01).

Concerning failure modes, bulk of the failures witnessed were of adhesive kind (ranging between 80–100% for β-TCPA-5%-NTC, β-TCPA-5%-TC, EA-NTC, and EA-TC), followed equally by cohesive and mixed-type and failures (not exceeding 20% of the total failures, observed only for the EA group) (Table 1). None of the failures observed for the β-TCPA-5% group were of cohesive or mixed type.

### 3.4. FTIR and DC Analysis Outcomes

**Figure 5:**
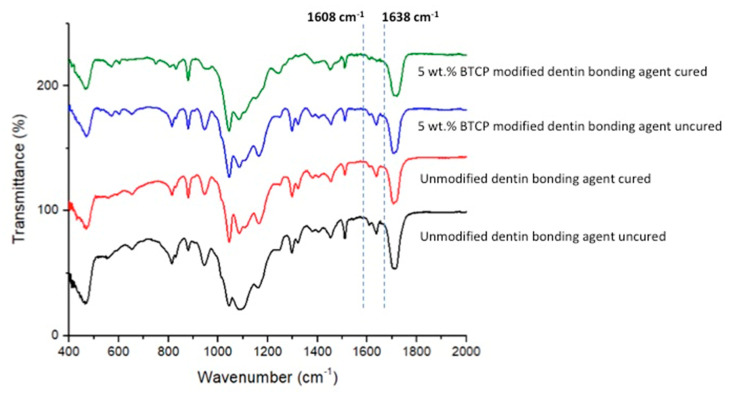
FTIR spectrum of polymerized and unpolymerized unmodified (EA) and βTCP nanocrystal modified adhesive. The degree of conversion was calculated by estimating the changes in peak height ratio of the absorbance intensities of aliphatic C=C peak at 1638 cm^−^^1^ and that of an internal standard peak of aromatic C=C at 1608 cm^−^^1^ during polymerization, in relation to the uncured adhesive as indicated by dotted lines.

The demonstrative FTIR spectra of cured and uncured EA and β-TCPA-5% groups were gathered and combined in Figure 5. The DC was appraised by estimating the discrepancies in peak height ratio of the absorbance intensities of aliphatic C=C peak at 1638 cm^−^^1^ and that of a standard inner peak of aromatic C=C at 1608 cm^−^^1^ while curing, as associated with the uncured adhesive as identified by the scattered lines (Figure 5). Concerning DC investigation, the highest DC was appreciated for EA (42.8 ± 3.1) followed by β-TCPA-5% group (38.3 ± 7.6) Table 2. No statistically significant results (*p* > 0.01) were witnessed upon the comparison of the DC values of the two adhesive groups. 

## 4. Discussion

Based on the study outcomes, our hypothesis was partially accepted as the presence of β-TCP nanoparticles enhanced the bond strength of the adhesive. The hypothesis was partially rejected, and we observed a lower DC for the β-TCPA-5% group compared to the EA. The inclusion of bioactive inorganic fillers can boost the mechanical properties of dentin adhesives [6]. The presence of essential remineralization ions like calcium and phosphate ensures that the dentin adhesive is capable of remineralizing the adhesive-dentin bond [24]. TCP contains calcium and phosphate ions and exists in two distinct crystal phases: α and β phases. Comparing these polymorph phases, β-TCP is relatively less soluble and synthesized at a lower temperature than its counterpart [25]. Additionally, the β-TCP nanoparticles are highly biocompatible and biodegradable in physiological environments [26]. Considering all these advantageous properties of these fillers, we were encouraged to incorporate β-TCP nanoparticles in our EA and evaluate their influence on various properties of the adhesive.

Our filler nanoparticles revealed irregular morphology, and many of these particles were found in agglomerated form (Figure 1A,B). Our results agree with Ruiz-Aguilar et al., who also demonstrated that β-TCP nanoparticles possess agglomerated morphology [27]. Researchers should note that due to the unpredictable morphology of β-TCP nanoparticles after synthesis, it is not easy to envision the exact shape of these particles post-synthesis. The micro-Raman spectra (Figure 2) of our filler revealed characteristic peaks associated with β-TCP nanoparticles, similar to an earlier study conducted by Arbez et al. [28].

Our β-TCP-5% adhesive validated appropriate dentin interaction, which was demonstrated by the formation of resin tags of varying depths (Figure 3B). These resin tags were comparable to the EA (unmodified with β-TCP nanoparticles) (Figure 3A). Although the measurements to assess the depth of the resin tags were not performed in our study, it should be noted that penetration depth of resin tags in the dentinal tubules does not significantly affect the strength of the bond and integrity of the adhesive [29]. The EDX mapping in our study verified the presence of two remineralizing ions (calcium and phosphate) in the β-TCP adhesive (Figure 4A,B). The presence of these two ions warrants the remineralization of the adhesive-dentin bond, improving the longevity of the composite restoration [30].

We utilized μTBS testing in our study to examine the bond strength of both adhesive groups. A former study suggested that when the adhesives are reinforced with inorganic fillers, their wt.% concentration should not exceed >10%, as this could reduce their bond strength due to a subsequently intensified viscosity [31]. Shadowing this suggestion, our adhesives were not added with >5 wt.% of β-TCP nanoparticles. Our μTBS results were inclined in favor of β-TCP-5% adhesive, as for both TC and non-TC samples, more μTBS values were witnessed as compared with the controls. Earlier, Garcia et al. incorporated TCP nanofillers in their adhesive and reported a higher bond strength than the controls [32]. Our results echoed similar findings, and we also noticed an improved bond strength of our β-TCP nanoparticles containing adhesive. Previously, Noorani et al. recommended that materials enclosing remineralizing ions can release them intermittently [33], and this property could have caused an increased μTBS for the β-TCP-5% group, as seen in our study. Another conceivable reason to explain this result could be that materials with nano-remineralizing ions can biomineralize with collagen fibers of dentin [8], resulting in increased remineralization and enriched bond integrity, as detected in our study.

In the present study, most of the interfacial failures witnessed were of adhesive type. The adhesive failure arises due to the loss of adhesion, and fractures are not observable in resin or dentin [34]. This type of failure is commonly seen in the adhesives containing fillers [24,30], and no single unanimous reason is present in the literature to explain their occurrence. In order to provide the adhesive with a similar dynamic and aggressive challenge as seen in the oral cavity, we used TC to age the samples and then tested their μTBS. According to the ISO standard number 11405, TC of dental materials within a temperature range of 5 to 55 °C is appropriate to age them for a limited period of time [35]. Several previous similar studies have also verified that the bond strength decreases after aging [36,37]. Our results are in conformity as a decreased μTBS was also observed for the two adhesive groups after aging.

The DC analysis in our study demonstrated a higher DC for the EA group as opposed to the β-TCP-5% group. Certain former studies have established that although the integration of inorganic filler particles strengthens the bond strength of the adhesive, it affects its DC and a lower DC is appreciated [30,38]. Our results are in agreement with these studies as a higher DC for the EA group was observed. A higher DC is exceedingly required as it certifies that a satisfactory number of monomers are polymerized [39], consequently reducing the chances of nanoleakge and secondary caries development. A reasonable explanation of this finding could be that due to the opaque nature of the inorganic fillers, possibly the curing light could not penetrate enough, thus causing providing an obstacle in the satisfactory conversion of monomers into polymers, resulting in a low DC [40].

Although the findings of our study are encouraging, they should be cautiously interpreted. The addition of β-TCP nanoparticles in the adhesive led to an appreciation in its μTBS and verified suitable dentin interaction. However, a reduced DC was observed for β-TCP-5% adhesive. Therefore, future studies involving different β-TCP filler concentrations should be conducted to establish the influence of various filler concentrations on other mechanical properties of the adhesive.

Our study had certain limitations. One limitation was its in vitro nature as a real in-vivo oral environment could be dynamic, offering multiple challenges to the adhesive material. The other limitation was that we only analyzed one concentration of β-TCP filler nanoparticles (5 wt.%). Incorporation of other concentrations of this filler could help in establishing a more precise narrative of the impact of their inclusion on different properties of the EA.

## 5. Conclusions

The adding of β-TCP nanoparticles led to a surge in the adhesive’s μTBS. The incorporation of β-TCP filler particles resulted in a suitable dentin interaction, seen in the form of hybrid layer and resin tag formation. Nonetheless, a decreased DC was observed for the β-TCP-5% adhesive. Further studies designed to explore the impact of various filler concentrations on the mechanical properties of the adhesive are warranted. Our new β-TCP nanoparticles containing EA have the potential to be used in vivo and can offer various advantages (including improved bond strength) that can directly increase longevity of the restoration.

## Figures and Tables

**Table 1:** Means and SD for microtensile bond strength and failure modes among the study groups.

	μTBS (MPa) (Mean ± SD)			Failure Mode Analysis (%)		
Group (*n* = 10)	NTC	TC	*p*-Value *	Adhesive	Cohesive	Mixed
β-TCPA	34.11 ± 3.46 ^a,A^	-	<0.01	100	0	0
	-	30.38 ± 3.66 ^a,A^		100	0	0
EA	28.07 ± 2.60 ^a,B^			80	10	10
	-	20.94 ± 3.37 ^b,B^		80	10	10

β-TCPA: beta-tricalcium phosphate adhesive, EA: experimental adhesive, * ANOVA. Dissimilar small alphabets in rows of the same adhesive, denote statistical difference (*p* < 0.01). Dissimilar capital alphabets in the same column denote statistical difference (*p* < 0.01).

**Table 2:** Degree of conversion in percentage of the 5 wt.% nano-β-tricalcium phosphate modified experimental adhesives and control.

Group	Degree of Conversion (Mean ± SD)	Tukey
EA	42.8 ± 3.1	A
β-TCPA	38.3 ± 7.6	B

Dissimilar uppercase letters indicate statistical significance.

## Data Availability

Data of the study are available on request form the corresponding author.
